# Preliminary Study and Pre-Validation in Portugal of New Farmers’ Mindfulness and Life Satisfaction Scale (FMLSS)

**DOI:** 10.3390/healthcare13091027

**Published:** 2025-04-29

**Authors:** Artur Morais, Raquel P. F. Guiné, Cristina A. Costa, Cátia Magalhães

**Affiliations:** 1 ESAV—Escola Superior Agrária de Viseu, Estrada de Nelas, Ranhados, 3500-606 Viseu, Portugal; arturfsmorais2001@gmail.com (A.M.); amarocosta@esav.ipv.pt (C.A.C.); 2 CERNAS—IPV Research Centre, Polytechnic University of Viseu, Campus Politécnico, 3504-510 Viseu, Portugal; 3 ESEV—Escola Superior de Educação, Rua Maximiano Aragão, 3504-501 Viseu, Portugal; cmagalhaes@esev.ipv.pt; 4 CI&DEI Research Centre, Polytechnic University of Viseu, Campus Politécnico, 3504-510 Viseu, Portugal

**Keywords:** farmer, family farming, farmers’ stress, rural development, mental well-being

## Abstract

**Background/Objective:** Besides the common risks associated with agriculture, recently, there has been growing concern about the impact of agriculture on farmers’ mental health, due to high stress levels, depression, anxiety, and increasing rates of suicide, especially complex considering that many of these farmers are older people. The potential of the practice of mindfulness to minimize mental health problems and improve people’s sense of well-being has been studied in recent decades, although there is a dearth of literature related to farmer populations. This study aimed to correlate the presence of mindfulness traits with general life quality and well-being and assess the levels of mindfulness and life satisfaction among family farmers, as well as to evaluate which characteristics might be associated with them. **Method:** The sample was composed of 30 farmers from the region of Viseu—Portugal, who were randomly selected for a survey consisting of an adaptation of the Mindful Attention Awareness Scale (MAAS) and the Satisfaction with Life Scale (SWLS), with some new items specific to the context of agriculture. A proposed Farmers’ Mindfulness and Life Satisfaction Scale (FMLSS) was validated through factor analysis and internal reliability analysis. **Result:** The results showed a relatively high average score for the 10 items of the mindfulness scale (4.23 ± 0.56) and the global sum of scores for the 5 items of the life satisfaction scale (26.67 ± 4.76). Factor analysis revealed six factors, globally explaining 77% of the variance, with values of alpha varying from 0.640 to 0.874. The FMLSS was validated with 19 items of the 20 initially considered (α = 0.672). Cluster analysis revealed two typologies of participants, “Pleased” and “Accommodated” family farmers. These two clusters had global values for the FMLSS of 5.19 ± 0.51 and 4.37 ± 0.59, with the higher value obtained for the “Pleased” family farmers, who were mostly of male gender and worked more hours per week and whose agricultural activities had higher significance for their family income. **Conclusions:** Overall, we observed a relatively high level of mindfulness and satisfaction with life among family farmers. This suggests the importance of future research on mental health among family farmers.

## 1. Introduction

Recent data suggest that mental health and suicide crises faced by farming communities are becoming increasingly concerning as globalization, trade liberalization, financial deregulation, resource scarcity, rural depopulation, and climate change advance at great speed [1]. Hence, the risk of developing mental illnesses/disorders and the increasing suicide rates among farmers has emerged as a priority in the context of agricultural environments [2].

According to the most recent Agricultural Census [3], most farms in Portugal are managed by individual producers (94.5%), most of them being operated in a family farm model.

Although it is portrayed as a healthy and peaceful lifestyle, farming is the professional sector in Europe with the fourth highest mortality rate (11.6%), particularly from work-related accidents, after the construction (22.5%), manufacturing (14.7%), and transportation and storage (16.7%) sectors [4].

Farmers are constantly being subjected to various health hazards, which are becoming more prevalent due to various agricultural, technological, industrial, and socio-economic factors, such as prolonged exposure to pesticides and other chemicals, physically demanding work and long work days, and exposure to various weather conditions [1,5]. However, the high mortality rate is not only due to the range of physical risks [2]. Suicide is the second leading cause of death in young farmers in Spain, after work-related accidents, but also in older and/or retired farmers, as well as their spouses [6]. In the United States, male farm owners have suicide rates around twice as high as the general population [7].

In Portugal, this subject is poorly documented. Therefore, potential factors responsible for this increase, or even strategies to mitigate it, have not yet been explored or identified. Although there are no specific data regarding farmers, Portugal had a documented suicide rate in 2019 of 11.5 per 100,000 inhabitants (5.7 and 17.9, respectively, for women and men) [8]. In 2021, the suicide rate in Portugal was 8.9 deaths per 100,000 inhabitants, with a higher incidence in males, whose suicide rate was four times higher than in females [9]. Associated with this evidence, there are high levels of stress, depression, and anxiety [10] and greater susceptibility to other psychological symptoms, including burnout, hopelessness, and loss of self-esteem [11].

Recently, there has been increasing concern about and interest in understanding the impact of agriculture on farmers’ mental health [1]. Even in the general population, chronic stressors such as financial, family, and environmental factors have a major influence on well-being and mental health. Stress is often associated with an increase in the prevalence of mental disorders, such as depression and anxiety [12], conditions that are also associated with a higher risk of mortality in adults in the general population [13]. Over the past 50 years, various social, political, and clinical bodies have endeavored to intervene specifically in the well-being and mental health of agricultural producers by developing programs aimed at addressing and fighting the root causes of stress [11].

Agriculture is an activity that generally requires long-term thinking and planning and is constantly subjected to unpredictable changes as a result of weather and market conditions [14]. According to Alma Jorgenson, a farmer from Minnesota who runs a mental health support program in rural areas, being focused on the present moment can help reduce anxiety and stress caused by the uncertainty of the future [15], something that is very much felt in agriculture. This state of full attention to the present moment, as well as emotions, feelings, sensations and the surrounding environment, is called mindfulness [16]. In this sense, mindfulness can be defined as attention and awareness of current experiences, characterized by non-judgmental observation and experiencing the present moment [17,18].

Mindfulness can be practiced or trained (and is not necessarily a permanent way of being) [16], but many people naturally possess some of its characteristic traits. Individuals who display more mindfulness traits tend to have a higher degree of life satisfaction [19]. Although this concept has not yet been thoroughly explored in the farming context, several studies on other areas have shown various benefits of its application, such as reduced stress, improved self-esteem, an improved immune system and creativity, increased resilience, self-control, and self-regulation [16,20,21,22,23]. Additionally, it has been increasingly associated with greater satisfaction with life [20,21]. The degree of satisfaction with life has been widely recognized as one of the key indicators of well-being, particularly mental well-being [24], and a higher degree of well-being is also associated with a lower risk of depression symptoms and, consequently, lower suicide rates [13].

The identification and analysis of mindfulness traits and states, as well as their correlation with physical and mental well-being and life satisfaction, can be an important tool to help develop new, appropriate, and efficient plans to combat the main factors responsible for the increase in stress, mental disorders, and suicide rates among farmers. Hence, the main aim of this study was to help identify traits of mindfulness, life satisfaction, and well-being in Portuguese farmers, which can help support the development of programs to improve farmers’ mental health. Specifically, the objectives were to assess the mindfulness traits present in family farmers, characterize them, and understand their impact on well-being, life satisfaction, and agriculture. To achieve this mail goal, a new scale was developed and validated, in order to make it reliable for utilization in future studies. This scale was obtained in two complementary ways: (1) the adaptation of some questions from two existing instruments (Mindful Attention Awareness Scale [18] and Satisfaction with Life Scale [25]), after translation into Portuguese; (2) complementing this scale with some new original questions designed by the team of researchers, specifically to address the intended objectives, i.e., looking at the variables of interest considering the subjects of study—the farmers.

## 2. Materials and Methods

This is a pre-validation study, conducted on a limited sample recruited through convenience sampling, according to the suggestions of the municipalities involved in the study, conducted in the central region of Portugal. Although all validation methodologies applied were the same as those used to validate the scales, the limited sample size allowed for more limited conclusions. Still, this study focuses on evaluating the initial feasibility of the FMLSS, which, if pre-validation is successful, will allow for a later study dedicated to more formal validation.

### 2.1. Instruments

To identify the main mindfulness traits and assess farmers’ general satisfaction with life, a survey was carried out through a questionnaire that included the farmers’ sociodemographic characterization, a description of their farms, and scales for assessing mindfulness traits and satisfaction with life (Table 1):

1. A mindfulness scale consisting of 10 items, adapted from Brown and Ryan’s Mindful Attention Awareness Scale (MAAS) [18] and based on the work of Qiu et al. [26], who rewrote four of the statements so that they relate more to rural and farming daily life. The original MAAS consists of 15 items that measure the tendency to be attentive and aware of present experiences in daily life. The scale is structured to gauge how frequently individuals experience moments of awareness, concentration, and engagement with their current activities, thoughts, and feelings [18,27]. Participants rate the frequency of their experiences on a 6-point Likert scale ranging from “almost always” to “almost never”. The final score on the scale is obtained from the average sum of the scores on all the items. Higher mean scores indicate a greater level of or disposition towards mindfulness.
2. The Satisfaction with Life Scale (SWLS), developed by Diener et al. [25], consisting of 5 items to measure the individual’s judgment of their general satisfaction with life. Participants indicate the extent to which they agree or disagree with each of the five items on a seven-point scale ranging from 1—strongly disagree to 7—strongly agree. The final score is obtained by adding up the points obtained for all the answers given to the statements and comparing them with some benchmarks: 5–9, “extremely dissatisfied”; 10–14, “dissatisfied”; 15–19, “a little dissatisfied”; 20, “neither satisfied nor dissatisfied”; 21–25, “a little satisfied”; 26–30, “satisfied”; 31–35, “extremely satisfied”.
3. Five items to be analyzed individually following a seven-point scale like the SWLS, which were written in the context of this study, based on the literature review carried out (“Spending more time outdoors and in contact with nature makes me feel better”; “I tend to worry about what other people think about me or what I do”; “I often feel overwhelmed by the weight of responsibilities”; “Farming makes me feel good”; “I enjoy what I do, and I feel valued for it”).

**Table 1:** The items used in the questionnaire and their sources.

Scales	Items	Refs.
Mindful Attention Awareness Scale (MAAS)	(1) I am aware that my emotions during harvesting can influence my thinking and behaviour	[26]
	(2) When something unexpected happens during harvesting, I am aware of my emotional state	[26]
	(3) When something during harvesting doesn’t go well, I am aware of my inner frustration and restlessness	[26]
	(4) When the situation changes during the harvesting, I am aware of the thoughts and ideas that flashed across my mind	[26]
	(5) I find it difficult to stay focused on what’s happening in the present	[18,27]
	(6) It seems I am ‘running on automatic’, without much awareness of what I’m doing	[18,27]
	(7) I find myself doing things without paying attention	[18,27]
	(8) I find myself listening to someone with one ear, doing something else at the same time	[18,27]
	(9) I find myself preoccupied with the future or the past	[18,27]
	(10) I snack without being aware that I’m eating	[18,27]
Satisfaction with Life Scale (SWLS)	(11) In most ways my life is close to my ideal	[25]
	(12) The conditions of my life are excellent	[25]
	(13) I am satisfied with my life	[25]
	(14) So far I have gotten the important things I want in life	[25]
	(15) If I could live my life over, I would change almost nothing	[25]
New Proposed Items	(16) Spending more time outdoors and in contact with nature makes me feel better	o.a.
	(17) I tend to worry about what other people think about me or what I do	o.a.
	(18) I often feel overwhelmed by the weight of responsibilities	o.a.
	(19) Farming makes me feel good	o.a.
	(20) I enjoy what I do, and I feel valued for it.	o.a.

o.a. = own authorship.

### 2.2. Data Collection

The survey was administered to Portuguese farmers in the Portuguese language, so the items that were adapted from the Mindful Attention Awareness Scale [18] and Satisfaction with Life Scale [25] were first translated to the Portuguese language. For the translation, independent researchers carried out the procedure, and an expert in Psychology performed linguistic and content validation. On the other hand, the items that were newly created by the researchers were originally in the Portuguese language. This Portuguese version of the questions was the one that was submitted to the Ethics Committee for approval. In Appendix A, the original Portuguese version of the questions is provided, in the language that they were presented to the farmers.

The survey was designed to be administered in person to family farmers located in the central region of Portugal. The selection of the farmers was made according to local authorities of the municipality of Viseu, and the parish councils of the Viseu district appointed them. For this reason, the sample was a convenience sample recruited according the directives of the municipalities. The survey received approval from the Ethics Committee of the Polytechnic University of Viseu on 21 June 2023 (Reference 34/SUB/2023).

Only participants aged 18 years or older were included in the study, and the interviews were carried out between June and September of 2023. In the end, 30 farmers were interviewed in person, and they were informed that their participation was voluntary and anonymous. Anonymized data were retained for the study and then deleted. The participants could withdraw their consent and participation in the study at any time, even during or after completing the questionnaire. Additionally, farmers were encouraged to ask questions for clarification as much as needed during the interview.

### 2.3. Data Analysis

The data were analyzed using descriptive statistics, alongside techniques to identify possible relations between the items on the scales and the various sociodemographic variables.

For the MAAS and SWLS, the internal consistency of their items and the overall reliability of the scales were checked, as was the correlation index between the two scales.

An exploratory data analysis was carried out using principal component analysis (PCA). The MAS (Measure of Sample Adequacy) values were relatively low, with 7 values equal to or greater than 0.5, as was the KMO, with a value of 0.427. The result of Bartlet’s test of sphericity was significant (*p* < 0.001), so a principal component analysis (PCA) was carried out using the Varimax rotation method with Kaiser normalization. Variables with coefficients lower than 0.4 were excluded and considered in the factor with the highest weight. The number of factors extracted was 6, explaining 76.9% of the variance.

A hierarchical cluster analysis was carried out to identify groups of farmers with similar characteristics and differentiate typologies of family farmers. First, the appropriate number of clusters was determined (set at 4), and the solutions obtained were used to build the clusters. Five solutions were tested: “Centroid”, “Ward”, “Between groups”, “Within groups” and “Single linkage”. Of the 5 initial solutions tested, 3 of them converged on the same final solution (“Centroid”–“Between groups”–“Single linkage”). This initially resulted in four clusters, two of which consisted of just 1 case, and therefore, a new cluster structure was defined with only two clusters, one with 26 farmers and the other 4 farmers. Characterization of the cluster members was performed using crosstabs based on the sociodemographic, professional, and lifestyle characteristics of the participants.

Also, T-tests for independent samples were used to compare the means between groups, namely between clusters. The power of the test was calculated (1 − beta), which is the probability of rejecting the null hypothesis when it is false, i.e., it is the probability of avoiding a type II error. For power analysis and all other statistical tests, alpha was considered to equal 0.05.

All statistical tests were carried out at a 95% confidence level, and the statistical analysis was carried out using Microsoft Excel Office 365, XLSTAT 2023, and IBM SPSS 29.

## 3. Results

### 3.1. Sociodemographic Characteristics of the Sample

The participating farmers were from different parish councils of the Viseu district, the councils of Viseu and Castro Daire, with higher incidence in *Abraveses* (*n* = 6), *Coutos de Viseu* (*n* = 6), and *Parada de Ester* (*n* = 6). Other parish councils with fewer participants were *Rio de Loba* (*n* = 3), *Silgueiros* and *Repeses* (*n* = 2 each), and *Avelal*, *Cota*, *Gosende*, *Mundão*, and *Viseu* (one participant from each).

The average age of the 30 interviewed farmers was approximately 61 years old, varying from a minimum of 41 to a maximum of 84 years, with farmers being, on average, 60.53 ± 12.61 years. Males made up most participants, representing two thirds of the sample (Table 2). Households tended to be small, with more than half of them made up of fewer than three members, often limited to couples. Around 57% of these households included members over 60 years of age, while 23.3% had children under the age of 18, and only 10% lived with grandchildren. Regarding education level, two thirds had a primary school level of education, with only 23.3% possessing a university degree.

Table 3 presents the data on the professional characteristics of the farmers, area of the farms, and income.

As for professional occupation, 43.3% of participants were employed, followed by 40% who were retired and 10% who were unemployed. Furthermore, 30% of those who had other occupations performed physically demanding jobs, such as for example construction, metalworking, carpentry, among others, while 20% were domestic workers. The majority of farmers had a family history in agriculture, with 73.3% having parents who were or still are farmers. Regarding the time dedicated to agriculture, almost half of the farmers (43.3%) worked less than 20 h a week on their farms, many of them complementing it with other professions. The average size of the farms was 0.34 hectares, with the majority having less than 0.25 ha. In terms of monthly family income, most (56.7%) earned less than 1365 €, with 30% receiving between 665 and 865 €.

Table 4 shows the lifestyle characteristics of the participants in the study. When it comes to daily habits, most farming family members (53.3%) drank alcohol four or more times a week; slept 6 to 8 h per day (63.3%); did not participate in organizations, cultural associations, or events (70%); and did not even go to social places, like a cafe or a friend’s house, after work (60%).

### 3.2. Mindfulness and Life Satisfaction Scores Among Family Farmers

The internal consistency of the adapted MAAS, measured using Cronbach’s alpha (Table 5), was relatively low (0.40), indicating low internal consistency of the items. However, if items (1) (“I am aware that my emotions during harvesting can influence my thinking and behaviour”) and (9) (“I find myself preoccupied with the future or the past”) were removed, the Cronbach’s alpha obtained would increase to 0.60.

The items on the adapted MAAS with a lower average score were (9) (“I find myself preoccupied with the future or the past”) (2.77 ± 1.26) and (5) (“I find it difficult to stay focused on what’s happening in the present”) (3.47 ± 1.52).

Regarding the SWLS, the obtained Cronbach’s alpha was 0.80, which is considered good. Additionally, this is very close to the original value of the alpha for this scale (α = 0.82) [25].

In the SWLS the item with highest mean score was (14) (“So far I have gotten the important things I want in life”) (6.23 ± 0.96). On the other hand, the item with lowest mean score was (15) (“If I could live my life over, I would change almost nothing”) (4.57 ± 1.61).

It was further observed that 76.7% of the farmers gave the maximum score (seven) to item (16) (“Spending more time outdoors and in contact with nature makes me feel better”), resulting in a very high mean score (6.67 ± 0.70). About half of the respondents fully agreed with the statement of item (19) (“Farming makes me feel good”), resulting also in a high average score (6.03 ± 1.35). The next highest mean score of the individual items was for item (20) (“I enjoy what I do, and I feel valued for it”) (5.30 ± 1.42), with 76.7% of the participants showing that they enjoyed what they did and felt valued for it. More than 63% of family farmers showed little or no concern for the opinion of others (17) (“I tend to worry about what other people think about me or what I do”) (2.63 ± 1.47), but 30% agreed that they felt pressured by the weight of responsibilities (18) (“I often feel overwhelmed by the weight of responsibilities”) (3.70 ± 1.37).

The global scores for the participants in both scales were calculated. For the mindfulness scale, MAAS, the global score was calculated as an average from the mean scores of all 30 family farmers to all 10 items of the scale (each item ranges from 1 to 6, and the global score is also from 1 to 6). The higher the score, the greater the level of or disposition towards mindfulness. Around half of the farmers had a mean score close to 4, and one-third had a score close to 5. The calculated global MAAS score for family farmers varied from a minimum of 3.1 to a maximum of 5.4, and had a mean value of 4.23 ± 0.56.

Regarding the Satisfaction With Life Scale (SWLS), the average score was obtained from the scores of all 30 family farmers, which were, in turn, calculated as the sum of the scores of all five items of the scale. Each item ranges from 1 to 7, and the global score ranges from 5 to 35. The final scores were categorized into benchmarks that range from “extremely dissatisfied” (score between 5 and 9) to “extremely satisfied” (Score between 31 and 35). Around one-third of the family farmers were not very satisfied with life, and about a half were satisfied or extremely satisfied with life. The rest were neither satisfied nor dissatisfied. The calculated global SWLS score for the family farmers varied from a minimum of 13 to a maximum of 35, and the mean value was 26.67 ± 4.76.

The correlation coefficient between the two scales, MAAS and SWLS, was also calculated to investigate whether there was an association between these two. Given the small sample size (*n* = 30), a non-parametric correlation was calculated using Spearman’s coefficient. The correlation coefficient was −0.043 (*p*-value = 0.822).

### 3.3. Validation of the New Farmers’ Mindfulness and Life Satisfaction Scale (FMLSS) Through Factor Analysis

Table 6 shows the results of the factors analysis of the items in the proposed 20-item Farmers’ Mindfulness and Life Satisfaction Scale (FMLSS). For the input weight of the variables into the factors, only those with coefficients of 0.4 or over were considered, corresponding to 16% of the variance explained. Also, each variable was considered in the factor where it had a higher weight. The final number of factors extracted was six, globally explaining 76.9% of the variance. Note that because the items used in the questionnaire were from different scales and with different scoring intervals, before proceeding to the factor analysis, standardization of the items was performed to ensure that they all aligned with the 7-point scale. This procedure covered items (1) to (10) so that all 20 items were in the same measuring interval from a minimum score of 1 to a maximum score of 7. Nevertheless, while the scale for questions (1) to (10) is a frequency scale varying from 1 = almost always to 7 = almost never, the scale for items (11) to (20) is an agreement scale varying from 1 = strongly disagree to 7 = strongly agree.

Regarding the exploratory analysis of the items, the internal consistency of factor F1 was very good (α = 0.874). Given the values of the loadings, the first factor (F1) showed a strong association with items (12) (“The conditions of my life are excellent”), (13) (“I am satisfied with my life”), and (14) (“So far I have gotten the important things I want in life”) of the SWLS and item (16) (“Spending more time outdoors and in contact with nature makes me feel better”) of the newly proposed items, all with values higher than 0.7.

The second factor (F2) showed a relation between items (2) (“When something unexpected happens during harvesting, I am aware of my emotional state”), (3) (“When something during harvesting doesn’t go well, I am aware of my inner frustration and restlessness”), and (10) (“I snack without being aware that I’m eating”), with α = 0.767. With the exclusion of item (10) (“I snack without being aware that I’m eating”), the relation between the variables was even closer, with α = 0.839.

In factor F3, it was possible to observe a strong relation between items (1) (“I am aware that my emotions during harvesting can influence my thinking and behaviour”), (11) (“In most ways my life is close to my ideal”), (15) (“If I could live my life over, I would change almost nothing”), and (19) (“Farming makes me feel good”), with good internal reliability (α = 0.703).

For factor F4, there was low consistency in the scores given to items (6) (“It seems I am running on automatic, without much awareness of what I’m doing”), (8) (“I find myself listening to someone with one ear, doing something else at the same time”), (9) (“I find myself preoccupied with the future or the past”), and (20) (“I enjoy what I do, and I feel valued for it”) (α = 0. 173). Nevertheless, in this factor, the internal consistency would increase to α = 0.640 if item (9) were excluded.

Factor F5 consists of only two items, (5) (“I find it difficult to stay focused on what’s happening in the present”) and (7) (“I find myself doing things without paying attention”) (α = 0.686).

Finally, for factor F6, there was also an association between items (4) (“When the situation changes during the harvesting, I am aware of the thoughts and ideas that flashed across my mind”), (17) (“I tend to worry about what other people think about me or what I do”), and (18) (“I often feel overwhelmed by the weight of responsibilities”) (α = 0.658). However, the internal consistency of the factor would increase to α = 0.672 if item (4) were deleted.

The global value of Cronbach’s alpha was 0.611, which can be considered acceptable based on the recommendation of Davis [28], according to whom a value over 0.5 is suitable for small groups of 25–50 individuals. However, by excluding the three items recommended based on the factors’ internal consistency, (4), (9), and (10), the global internal consistency of the scale increased to 0.55. Nevertheless, it was further observed that alpha still increased to 0.672 if two items, (4) and (10), were back on the scale, meaning that, despite these items not correlating so well with their factors (F6 and F2, respectively), they are still meaningful in the overall scale. Based on this evidence, the final validated FMLSS proposed contains 19 items, only excluding item (9) (“I find myself preoccupied with the future or the past”), and all in a measuring scale of seven points. In Appendix A is presented the final FMLSS and the corresponding recommended measuring scales.

### 3.4. Typology of Farmers Based on Cluster Analysis

Table 7 presents the mean scores for the 20 items used in the survey separately, plus the mean scores of the different scales (MAAS, SWLS, FMLSS), for the two sets of family farmers. Cluster analysis grouped the participants according to the similarity of their responses considering the six factors of the previously performed factor analysis.

Based on the results for both clusters, distinct traits of mindfulness and life satisfaction were observed, and it was possible to identify the basic typology of the farmers in each of the two groups. Cluster one, which comprised 87% of the family farmers, grouped the farmers showing higher satisfaction with life and their professional activity and also with higher trend for mindfulness. This cluster was named the “Pleased farmers”. On the other hand, cluster 2, including 13% of the family farmers, corresponded to individuals less satisfied with life and with a lower predisposition to mindfulness, but still with positive scores, so is was named the “Accommodated farmers”.

The “Pleased farmers” had a global MAAS score of 4.91 ± 0.67, with the highest mean scores for items (3) (“When something during harvesting doesn’t go well, I am aware of my inner frustration and restlessness”) (5.98 ± 1.16) and (1) (“I am aware that my emotions during harvesting can influence my thinking and behaviour”) (5.85 ± 1.61). Item (9) (“I find myself preoccupied with the future or the past”), although it had a low value showed, that worries are present (2.98 ± 1.27). The “Pleased farmers” showed a global SWLS score of 5.51 ± 0.84, with high mean scores for items (14) (“So far I have gotten the important things I want in life”) (6.15 ± 1.01), and (11) (“In most ways my life is close to my ideal”) (5.46 ± 1.17). Also, these farmers showed high values for items (16) (“Spending more time outdoors and in contact with nature makes me feel better”) (6.73 ± 0.53) and (19) (“Farming makes me feel good”) (6.27 ± 0.87). The global score of the FMLSS for the “Pleased farmers” was high (5.19 ± 0.51).

For cluster 2, the “Accommodated farmers”, the global scores obtained for the MAAS, SWLS, and FMLSS were 4.66 ± 0.65, 4.15 ± 1.05, and 4.37 ± 0.59, respectively. Items (1), (11), (15), and (20) had scores that were noticeably lower (1.90 ± 1.15, 3.25 ± 0.96, 1.75 ± 0.96, and 3.50 ± 2.38, respectively), while for items (2), (3), (9), and (14) the scores were higher (6.40 ± 0.69, 6.10 ± 1.15, 4.00 ± 2.86, and 6.75 ± 0.50, respectively).

The results in Table 7 indicate that for most items, the differences between clusters were not significant, with the exception of only items (1), (11), (12), and (15), while for the scales, significant differences were found in two out of three cases, for scales SWLS and FMLSS. The power of the test was variable from a minimum of 0.155 to a maximum of 0.963 (96.3%).

To better understand the trends observed for the two typologies of farmers, characterization of the clusters was carried out based on the sociodemographic and lifestyle characteristics of the farmers (Table 8).

The “Pleased farmers” (Cluster 1) were mostly male, aged under 59 years, and married, with households of one or two persons. Most had completed only basic education and had very small cultivated areas (under 0.25 ha) (Table 8). In general, these farmers had no professional training in agriculture and had obtained their agricultural knowledge from their parents, who had also been farmers. They were not full-time farmers—all had another job—but they dedicated 20 to 40 h a week to their farm, almost working a “double shift”. Most of them had a monthly income over EUR 1366, and farming made a residual contribution to their monthly family income. As far as habits were concerned, these farmers drank alcohol four or more times a week, slept 6 to 8 h a day, and had very few or no social habits (going to a café, a friend’s house, a social event, etc.) (Table 8). The small family farmers, despite having small cultivated areas, had their own houses on the property most of the time and could support their family. According to Zhang et al. [29], housing ownership plays a positive role in improving life satisfaction. According to Azima and Mundler [30], male and female farmers experience different degrees of professional satisfaction in short food chains to those usually associated with family farming. Female farmers value the increased autonomy and opportunities for development, while male farmers demonstrate higher concerns for care and ecology [30].

Regarding the “Accommodated farmers” (cluster 2), they had specific characteristics that were distinctive, in most cases, from those of the “Pleased farmers” (Table 8). The “Accommodated farmers” were mostly female and married. They had parents who were farmers and had very small farms (with cultivated areas below 0.25 ha). They spent fewer hours farming (less than 20 h), and their agricultural activities also provided residual monthly income. Like the “Pleased farmers”, the “Accommodated farmers” lived solitary lives, not engaging in social activities (Table 8). Liu et al. [31] investigated how to be a happy farmer by engaging in technology. They tested some factors which are well documented in the scientific literature for their correlations with happiness and life satisfaction (income and leisure), and how technology can impact well-being. They concluded that the adoption of agricultural mechanization led to increased income and free time to dedicate to leisure activities, thus contributing to increased happiness [31].

## 4. Discussion

### 4.1. Characteristics of the Farmers

More than half of the farmers revealed very low values of income, with one-third receiving between EUR 665 and EUR 865. For comparison, in 2021, the average monthly net income of a couple in Portugal, both of whom were employed, was approximately EUR 2022 [32], and in the municipalities of Viseu and Castro Daire, the values were around EUR 1752 and EUR 1310, respectively [33]. Notably, more than half of the respondents (53.3%) considered that agriculture contributed little to their family’s monthly income. For the fraction of family farmers in which agriculture was responsible for the majority of their family income, the situation resulted from the financial support that they received from governmental and European supporting measures. Although the majority of agricultural production was used only for self-consumption, and thus not accounted for in the family budget, the low economic return from agriculture could be a cause of stress in rural families. The inability to obtain income from agricultural production may force farming family members to work with a formal contract or look for another job, which doubles the workload and puts one’s well-being and quality of life at risk. To overcome some of these difficulties, alternative food networks are gaining growing relevance by promoting sustainable transitions of food production, retail, and consumption [34]. Multiple actors in the food supply system defend the role of community-based initiatives at the niche level, which evolve and spread innovative practices that directly benefit small and family farmers in the context of proximity [35,36].

During the interviews, some of the study participants reported that, despite not obtaining money for what they produced, the agriculture that they practiced was an economic, nutritional, and safe added value. They had the capacity to produce their food, often of higher quality than that for sale in big supermarkets, and in sufficient quantity to satisfy several families’ food needs throughout the year. The role of small family farms in the survival, financial support, and food security of families is readily acknowledged. A systematic review by Zeleke and Wordofa [37] revealed that cluster farming (groups of farmers with adjacent land to cover large areas with similar crops and practices) contributed to increased income, improved livelihoods, and reduced poverty for smallholder farmers, most of the time family farmers. Additionally, these smallholders managed their farms with rigor, invested their efforts in producing quality agricultural goods, and contributed to favorable environments and market access, facilitating the short-circuit trade of crops and agricultural products and bringing the producer and the consumer closer. Possible additional off-farm income for farming family members can generate opportunities for additional sources of income, thus contributing to the financial sustainability of their households [37,38].

Regarding the farmers’ daily habits, most drank alcohol four or more times a week and slept between 6 and 8 h per day. They did not participate in social activities, not even going to a cafe or a friend’s house, after work. These results indicate a lack of socialization and interaction with people outside the family circle, which can make the farmer feel alone or isolated. According to Zhang and Li [39], the Social Network Theory has emerged as a valuable instrument to analyze interdisciplinary issues among rural communities. Hence, some studies have focused on evaluating not only socioeconomic aspects but also the physical and mental health of residents in rural areas, such as small family farmers [39]. Malone et al. [40] suggest that farmers rely on informal mental health support from non-official actors, relying on family, friends, and peers, and that proper support must be designed to target farmers’ emotional needs and communication styles, often based on a low literacy level.

### 4.2. Mindfulness and Life Satisfaction Among Family Farmers

The reliability analysis of the adapted MAAS revealed low internal consistency of the items. Some possible factors that could explain this include a relatively small sample size, possible misadaptation of the scale considering the target audience, or the reduction from the 15 original items to only 10 coming from two different scales. The obtained value was significantly lower than the Cronbach’s alpha obtained in the original study (α = 0.81) [18]. However, by removing two items ((1) “I am aware that my emotions during harvesting can influence my thinking and behaviour” and (9) “I find myself preoccupied with the future or the past”), the Cronbach’s alpha increased to a value which could be considered acceptable according to Davis [28], who recommends an alpha above 0.5 for small groups of 25–50 individuals.

The items on the adapted MAAS with a lower average score were (9) (“I find myself preoccupied with the future or the past”) and (5) (“I find it difficult to stay focused on what’s happening in the present”). These refer to situations of difficulty in focusing attention on the present moment and work, one of the main traits of mindfulness. Farming can be a very repetitive activity, in terms of work and manual techniques, which not only influences physical tiredness but also mental fatigue, which can cause less focus and attention on what is being done in the present, an action which, in some jobs, can be dangerous. Jones et al. [41] states that farmers are generally confronted with substantial occupational stressors and are described as experiencing great rates of burnout, anxiety, depression, and suicide. The high levels of stress in farmers at multiple levels (personal, interpersonal, and cognitive) have a negative impact on their professional performance, including productivity and business success [41].

Regarding the SWLS, the obtained Cronbach’s alpha was 0.80, which is very close to the original value of the alpha for this scale [25], and lay within the usual values of alpha (between 0.70 and 0.82) [42]. Hence, this indicates a close relation between the items of the scale and good internal reliability [42].

In the SWLS, the item with highest mean score was (14) (“So far I have gotten the important things I want in life”), meaning that most farmers fully agree with the statement, attributing maximum a score of seven. Conversely, the item with lowest mean score was (15) (“If I could live my life over, I would change almost nothing”), which means that the participants are so pleased with their lives that they would not like to change it. Stahlmann-Brown et al. [43] highlights the role of personal control in well-being and life satisfaction among farmers. Feeling control over one’s life is an especially strong predictor of well-being. While financial stress could contribute to decreased life satisfaction among farmers, the locus of control positively contributes to increased satisfaction with life [44]. The locus of control is an individual’s belief about to what degree one can control and influence future events. When one believes that they have the power to determine their future through their personal actions, the locus of control is categorized as internal. On the other hand, it is categorized as external when future events are controlled by outside forces like luck, fate, or other people. According to Zhao et al. [45] there are intergenerational and gender differences in farmers’ satisfaction with life, with older farmers being more satisfied than younger ones, and women being more satisfied than men.

It was further observed that two-thirds of the farmers gave the maximum score to item (16) (“Spending more time outdoors and in contact with nature makes me feel better”), resulting in a very high mean value, meaning most of them fully agree that time spent outdoors and in contact with nature positively contributes to their well-being. A study by Fan et al. [46] investigated the well-being of senior farmers and reported that social interaction and outdoor space are critical factors, and the neighborhood environment is a pivotal predictor of life satisfaction. Higher average mean scores were obtained for items (19) (“Farming makes me feel good”) and (20) (“I enjoy what I do, and I feel valued for it”). According to Liu et al. [31], income and leisure are two important predictors of happiness among farmers. A high percentage of family farmers showed little or no concern for the opinion of others (17) (“I tend to worry about what other people think about me or what I do”), but one-third agreed that they felt pressured by the weight of responsibilities (18) (“I often feel overwhelmed by the weight of responsibilities”). Farmers are autonomous and make their own financial decisions, and thus, are under greater pressure to handle multi-faceted stressful situations not only in the management of their farm but also in their family’s inner circle [44].

The calculated global MAAS mean score for family farmers was high, which indicates a positive level of or disposition towards a state of mindfulness. Barajas and Garra [47] applied the MAAS to a few groups, namely students (MAAS score = 3.57), the general Spanish population (MAAS = 4.08), groups of patients with anxiety (MAAS = 3.76), and patients with depression (MAAS = 3.42). Although a direct comparison cannot be made (since the scale was adapted), the values obtained in the MAAS by Barajas and Garra [47] were always lower than those obtained in the present study (MAAS = 4.23). In addition, the average value obtained was also higher than that of the first studies by the creators of the scale, Brown and Ryan [18], who applied the scale to groups of university students (MAAS = 3.72), psychology students (MAAS = 3.85), and the general American population (MAAS = 3.97). Nevertheless, during the administration of the survey, most of the participants reported a great sense of worry about the past and especially the future, more so than in the work of Barajas and Garra [47]. This worry may be associated with certain stress factors such as unpredictable weather, financial problems, geographical and social isolation, etc. [48,49,50,51].

Regarding the Satisfaction with Life Scale (SWLS), the calculated global score for family farmers was relatively high, which indicates that, in general, the family farmers surveyed were “satisfied with life”. When compared with other studies where the SWLS was applied, the average value of farmers’ satisfaction with life (SWLS = 26.67) was very close to the average value obtained for a group of Spanish university professors (SWLS = 26.90) in the study by Landa et al. [52]. When compared with the group in the study by Silva et al. [53], carried out with young Brazilian adults, the farmers in this study had higher average scores on all the items of the scale. However, when compared to the teachers in Ahammed’s [54] study, they were, on average, more satisfied with life (SWLS = 29.04). The most significant difference was between the farmers in this study and another group of elderly farmers [55]. The elderly farmers were generally quite dissatisfied with life, with an average SWLS score of 14.08. Note that the five items of the SWLS were the same, but in the work of the elderly, the measuring scale used was five points and not seven points as in the original scale and in the present work with family farmers. In this way, the maximum score for the elderly farmers would be 25 and not 35 points. According to the author of [55], these results can be explained by the lack of knowledge regarding the use of communication technologies, such as cell phones and computers, which prevent some older farmers from interacting with other people or being aware of more efficient agricultural practices and techniques, contributing to social isolation and demotivation.

The correlation coefficient between the MAAS and SWLS showed that there was practically no correlation between the mindfulness scale and the life satisfaction scale, and it was not a significant correlation. This lack of relation indicates that variations in the scores obtained in the MAAS are not explained by variations in the scores obtained in the SWLS. A family farmer’s quality of and satisfaction with life does not seem to relate to higher or lower levels of mindfulness. It would be expected that there was a positive correlation between a higher level of mindfulness and greater satisfaction with life, given the information from the literature and other studies [18,19,20]. This difference could be related to the differences in the scales, such as different scoring intervals and methods of calculating the global scores, or due to other factors of a psychological nature. The fact of experiencing a state of “presence” by the family farmers may not be due to their satisfaction with life but due to a state of consciousness, highly influenced by the need to be in a state of alertness given the risks associated with agricultural activity. This state of alertness could possibly lead to a state of mindfulness that may not have to do with satisfaction but rather with concern. This uncertainty in agricultural activity is highly related with natural phenomena and disasters, as well as with extreme weather conditions that sometimes compromise the whole production of one or more crops [56,57,58,59].

### 4.3. Validation of the FMLSS

The exploratory factor analysis showed very good internal consistency of factor F1, which included items (12) (“The conditions of my life are excellent”, (13) “I am satisfied with my life”), (14) (“So far I have gotten the important things I want in life”) and(16) (“Spending more time outdoors and in contact with nature makes me feel better”). There appears to be a relation between the positive feelings resulting from spending time outdoors and in contact with nature and greater satisfaction with life. Isaac et al. [60] reviewed the measures for farmer well-being, and highlighted that while the translocal approach is common in conventional well-being frameworks, farmer expression does not appear to be a key issue in well-being assessments, although it is much needed as a way to center farmers’ values while establishing well-being indicators.

Factor F2 showed a close association between items (2) (“When something unexpected happens during harvesting, I am aware of my emotional state”), (3) (“When something during harvesting doesn’t go well, I am aware of my inner frustration and restlessness”), and (10) (“I snack without being aware that I’m eating”), which indicates possible proximity between items related with awareness of one’s emotional state, frustration, and restlessness in the event of something unforeseen occurring during work. Furthermore, by excluding item (10) (“I snack without being aware that I’m eating”), the association between the variables in the factor increased. Farmers in agricultural communities are subject to various stressors, both of an internal and an external nature, causing a huge burden for farmers, and this can eventually be overwhelming. As such, some members of agricultural communities can have compromised mental well-being [61].

Factor F3 included items (1) (“I am aware that my emotions during harvesting can influence my thinking and behaviour”), (11) (“In most ways my life is close to my ideal”), (15) (“If I could live my life over, I would change almost nothing”), and (19) (“Farming makes me feel good”), and showed good internal reliability, indicating a possible relation between a better quality of life and a greater awareness of the influence of emotions at work and the well-being caused by agricultural practice. Cognitive emotion is related with psychological stressors and well-being [62], and emotional regulation assumes a pivotal role in improving quality of life [63].

Factor F4 presented low consistency and included items (6) (“It seems I am running on automatic, without much awareness of what I’m doing”), (8) (“I find myself listening to someone with one ear, doing something else at the same time”), (9) (“I find myself preoccupied with the future or the past”), and (20) (“I enjoy what I do, and I feel valued for it”). However, for this factor, it was observed that removing item (9) would improve internal consistency significantly. This high increase in the value of alpha in the factor may justify the exclusion of item (9) from the FMLSS to improve internal reliability. These results include some aspects of the mindfulness component (items (6) and (8)), like doing things automatically without much thinking and performing multiple activities at the same time, with item (20) related to the pleasure of performing farming activities and feeling psychologically rewarded by it. This sense of enjoying one’s work facilitates the performance of tasks with pleasure in a kind of automated workflow. According to Bardach and Murayama [64], motivation is driven by a sense of feeling rewarded.

Factor F5 includes two items, (5) (“I find it difficult to stay focused on what’s happening in the present”) and (7) (“I find myself doing things without paying attention”), and presents a good internal consistency. These items indicate some limitations of the focus on the present and a lack of attention when performing some activities, thus carrying them out without giving them much thought. Farmers perform many repetitive tasks, and on a daily basis, which contributes to this disengagement in some of them. Different expectations towards farming may cause identity issues and decrease work-related well-being [65].

Factor F6 included items (4) (“When the situation changes during the harvesting, I am aware of the thoughts and ideas that flashed across my mind”), (17) (“I tend to worry about what other people think about me or what I do”), and (18) (“I often feel overwhelmed by the weight of responsibilities”). The initial internal consistency was moderate and would increase slightly if item (4) were deleted. This factor suggests a relation between feeling the weight of responsibilities, eventually associated with some uncertainty related to harvest, and the tendency of farmers to worry about outside opinions about themselves or their actions. The sense of being accepted by others strongly contributes to increased life satisfaction according to Wu et al. [66].

### 4.4. Characterization of Farmer Clusters

The cluster analysis revealed two groups of farmers, the “Pleased farmers” and the “Accommodated farmers”.

The “Pleased farmers” had relevant mindfulness traits present, with the highest mean scores for mindful items related to self-awareness of frustration resulting from work problems and the role of emotions during harvest in thoughts and behavior, meaning that they seldom experience these feelings. On the other hand, for these farmers, worries about the future and the past are sometimes present. The “Pleased farmers” showed a relatively high global SWLS score, strongly believing they had achieved what they wanted up to the present moment, and feeling strongly satisfied with life. Also, the “Pleased farmers” strongly believed that spending more time outdoors and in contact with nature were activities that made them feel better, and farming strongly contributed for their well-being. Finally, the global score of the FMLSS for the “Pleased farmers” was high. Farmer satisfaction is influenced by the source of income and spending, and by living status, and increased satisfaction greatly affects farmers’ happiness [67]. Also, Hong et al. [68] report a positive association between income and satisfaction with life among forest farmers.

For the “Accommodated farmers”, the global scores obtained for the MAAS, SWLS, and FMLSS were consistently lower compared with the “Pleased farmers”.

Items (1) (“I am aware that my emotions during harvesting can influence my thinking and behaviour”), (11) (“In most ways my life is close to my ideal”), (15) (“If I could live my life over, I would change almost nothing“), and (20) (“I enjoy what I do, and I feel valued for it“) were those where the scores were noticeably lower. Conversely, for items (2) (“When something unexpected happens during harvesting, I am aware of my emotional state”), (3) (“When something during harvesting doesn’t go well, I am aware of my inner frustration and restlessness”), (9) (“I find myself preoccupied with the future or the past”), and (14) (“So far I have gotten the important things I want in life“), the scores were higher in comparison to those of the “Pleased farmers”. These results highlight that the “Accommodated farmers” are frequently aware of the role of their emotions, they do not feel that their life is close to what they idealized, they would change a lot if they had the chance to live their lives all over again, and they do not much enjoy what they do and do not feel enough valued for it. On the other hand, the “Accommodated farmers” are not alert to their emotional state and frustration in cases of harvest emergencies, they tend to not worry about the future or the past, and they strongly feel that they have already achieved their goals in life. Emergencies and events that compromise agricultural production, like those derived from severe climatic conditions and climate change, must represent opportunities for farmers to adapt to such conditions with minimal impact [69]. Nevertheless, the stress induced by such uncertainty is a factor affecting life satisfaction and relaxation felt in the present, as derived from the principles of mindfulness.

These results provide valuable insights into how to address the problems of mental health, well-being, and life satisfaction among farmers. This can help make better interpretations in the future by applying the pre-validated scale on a large scale among the Portuguese farmer population. The results of this work allowed us to understand family farmers’ way of life and their pragmatic view of their day-to-day lives, as well as to understand their difficulties and the things they value most in life. This knowledge proved essential in outlining the context and importance of further research on mental health among family farmers in Portugal. Based on some items of the MAAS, we observed a relatively high level of mindfulness among family farmers, and based on the items of the SWLS, it was concluded that the family farmers in this study are generally satisfied with life.

These results allowed us to validate the Farmers’ Mindfulness and Life Satisfaction Scale (FMLSS), adapted for farmers, composed of 19 items, which showed good internal reliability on a sample of Portuguese family farmers. Although the population surveyed showed certain homogeneity in their responses to the items on the scales, it was possible to identify two types of family farmers, Pleased and Accommodated farmers. The main difference between these two groups lies in the feeling of appreciation and well-being caused by farming, with much different scores relating to satisfaction with life and to items added specifically for the context of farming, namely “Farming makes me feel good” and “I enjoy what I do, and I feel valued for it”. The major differentiating characteristic between these two groups include gender (more male farmers in the Pleased group and more female farmers in the Accommodated group), weekly hours of farming activities (fewer hours for the Accommodated farmers than for the Pleased farmers), and the relevance of agricultural activities for family income (lower for Accommodated farmers).

## 5. Conclusions

This work showed some valuable results, regarding both mindfulness as well as life satisfaction among family farmers. These include the following:

- Farmers feel good by spending more time outdoors and in contact with nature;
- Farmers appreciate and recognize that they have already obtained the important things they want from life;
- Farmers feel good in their farming activities;
- Most farmers can be categorized as pleased with life;
- Few farmers fall into the cluster of Accommodated farmers;
- Pleased farmers showed higher scores in the Mindful Attention Awareness Scale;
- Pleased farmers also revealed higher scores in the Satisfaction with Life Scale;
- Validation of the Farmers’ Mindfulness and Life Satisfaction Scale resulted in a set of 19 out of 20 initial items tested.

## 6. Future Work and Limitations

Although the obtained results are of importance, now that the FMLSS has been validated, it would be valuable to extend this work to other farmers from other regions in Portugal, and eventually not only family farmers, and investigate the possible differences between family farmers and other groups of farmers. In the future, it would also be interesting to apply the scale in different countries to compare between farmers from different social contexts and geographic regions.

Validation at a larger scale would be a valuable tool to conduct further investigations in this line of work. Items like “I find myself doing things without paying attention”, “I find myself listening to someone with one ear, doing something else at the same time”, or “I snack without being aware that I’m eating” could be further subjected to a deeper analysis to confirm their true assessment of life satisfaction for farmers. This, in the future, would be a possible complementary study for these items as well as others.

The lack of information about mindfulness and life satisfaction for Portuguese farmers in the scientific literature turned out to be a major challenge in this work, but it also provided an opportunity to undertake this innovative study. Although several studies have been carried out in countries such as the United States, Australia, and England on the phenomena of increased suicide rates and symptoms of depression and anxiety among farmers, the reality is that in Portugal, data of this nature are scarce or even non-existent. The stress and risk factors for mental health in farmers presented in foreign studies may not be the same as or relevant to the reality of family farming in Portugal. The lack of or difficulty in accessing national studies that have used the same scales as this work makes it difficult to compare the results obtained with those of other groups, such as the general Portuguese population or people with other professions in Portugal. However, these limitations create a relevant area in the Portuguese agricultural scenario, and particularly in the family farming area, to be further explored and invested in the future. To that end, this work seeks to be a bibliographical and methodological basis for future work in that area.

The possible relations between several sociodemographic factors, as well as environmental or behavioral aspects, also constitute an important line of future development, since these relations or associations have not yet been explored. Studies show that many factors impact well-being, and possible relations between intertwining factors are of interest to better understand the mental health of farmers.

Although all the questionnaires were delivered and completed in person to assist farmers with any questions and ensure they understood the 20 items, there were some constraints. The MAAS was adapted to be shorter and more relevant to daily farming, which was beneficial for the farmers. However, the interview-like format of the questionnaire and the need to still explain items in simpler terms may have led some individuals to respond with what they thought was “the right answer” rather than reflecting their true experiences, therefore possibly introducing some bias.

Based on the results of this study, some pertinent questions arise for future study, namely pertaining to the influence of schooling, alcohol consumption, socialization and isolation, weekly workload, and average monthly income on mindfulness traits, life satisfaction, and well-being. In this study, the participants surveyed tended to have a low level of education, a high workload, and few social habits, so it would be interesting to compare them with other family farmers with opposite characteristics in these variables.

The importance of an approach guided by professionals is pivotal to helping design preventive measures to promote mental health and increase mental health literacy among farmers. This type of approach could allow a more detailed understanding of the stress factors affecting well-being and life satisfaction, and provide crucial insights for the effective implementation of interventions, including stress management measures, job security, risk management and psychological support. These types of studies allow us to assess the impacts of mindfulness practices on life satisfaction and mental health, aimed at improving the well-being of Portuguese farmers.

The Portuguese ministries of Agriculture, Social Security, and Health, should work together to plan interventions aimed at, based on evidence provided by scientific studies, effectively improving the mental health status of those who have to face difficult stressful situations, which tend to escalate as climate change evolves and extreme climatic phenomena become more frequent and cause more and more damage to their crops.

## Figures and Tables

**Table 2:** Sociodemographic characteristics of the participants.

Variables	Groups	Percentage (%)
Gender	Male	63.3
	Female	36.7
Size of the household	<3 persons	53.3
	≥3 persons	46.7
Marital status	Married/living together	90.0
	Divorced/separated	6.7
	Widowed	3.3
Education level	Basic (1st cycle—up to 4th year)	23.3
	Basic (2nd cycle—5 to 6th year)	40.0
	Basic (3rd cycle—7 to 9th year)	3.3
	Secondary (10 to 12th year)	10.0
	University degree	23.3

**Table 3:** Professional characteristics of the farmers, area of the farms, and income.

Variables	Groups	Percentage (%)
Parents were/are farmers	Yes	73.3
Has professional training in agriculture	Yes	16.7
Is a fulltime farmer	Yes	36.7
Has always been a farmer	Yes	16.7
Job situation	Employed	43.3
	Unemployed	10.0
	Retired	40.0
	Other	6.7
Type of work (other than farmer)	Manual/physically demanding	30.0
	Cleaning/domestic	20.0
	Driver	10.0
	Teaching	20.0
	Social/communication	20.0
Area of the farm	[0; 0.25] ha	63.3
	]0.25; 0.5] ha	6.7
	]0.5; 0.75] ha	10.0
	]0.75; 1] ha	20.0
Weekly hours of farm work	<20 h	43.3
	20–40 h	43.3
	40–60 h	10.0
	>60 h	3.3
Monthly family income	EUR 665–865	30.0
	EUR 866–1065	10.0
	EUR 1066–1365	16.7
	EUR 1366–1665	10.0
	EUR 1666–1995	16.7
	EUR >1996	16.7
Relevance of agriculture for family income	Residual (<25%)	53.3
	Low (25–50%)	36.7
	Medium (50–75%)	3.3
	High (>75%)	6.7

**Table 4:** Lifestyle characteristics of the farmers.

Variables	Groups	Percentage (%)
Frequency of consumption of alcoholic drinks	Never	6.7
	≤1 time/month	10.0
	2–4 times/month	10.0
	2–3 times/week	20.0
	≥4 times/week	53.3
Hours of sleep/day	6–8 h	63.3
	8–10 h	33.3
	>10 h	3.3
Participation in organizations, cultural associations, or events	None	70.0
	1–3 times/week	30.0
Go to associations for social engagement after work	None	60.0
	1–3 times/week	30.0
	4–6 times/week	10.0

**Table 5:** Mean scores for the scale items and internal reliability analysis.

Scale (Alpha) ^1^	Item	Mean ^2^	SD ^2^
MAAS—Mindful Attention Awareness Scale (α = 0.40)	(1) I am aware that my emotions during harvesting can influence my thinking and behaviour	4.60	1.69
	(2) When something unexpected happens during harvesting, I am aware of my emotional state	5.03	1.11
	(3) When something during harvesting doesn’t go well, I am aware of my inner frustration and restlessness	5.17	0.93
	(4) When the situation changes during the harvesting, I am aware of the thoughts and ideas that flashed across my mind	4.63	1.38
	(5) I find it difficult to stay focused on what’s happening in the present	3.47	1.52
	(6) It seems I am ‘running on automatic’, without much awareness of what I’m doing	3.83	1.46
	(7) I find myself doing things without paying attention	4.03	1.40
	(8) I find myself listening to someone with one ear, doing something else at the same time	3.90	1.68
	(9) I find myself preoccupied with the future or the past	2.77	1.26
	(10) I snack without being aware that I’m eating	4.83	1.59
SWLS—Satisfaction with Life Scale (α = 0.80)	(11) In most ways my life is close to my ideal	5.17	1.34
	(12) The conditions of my life are excellent	5.10	1.11
	(13) I am satisfied with my life	5.60	1.25
	(14) So far I have gotten the important things I want in life	6.23	0.96
	(15) If I could live my life over, I would change almost nothing	4.57	1.61
Newly Added Items	(16) Spending more time outdoors and in contact with nature makes me feel better	6.67	0.70
	(17) I tend to worry about what other people think about me or what I do	2.63	1.47
	(18) I often feel overwhelmed by the weight of responsibilities	3.70	1.37
	(19) Farming makes me feel good	6.03	1.35
	(20) I enjoy what I do, and I feel valued for it.	5.30	1.42

^1^ Cronbach’s alpha. ^2^ The mean and standard deviation of the scores calculated from all participants for each of the scale items. For the MAAS, the items and corresponding mean values vary from 1 to 6, whereas for the SWLS and the Newly Added Items, the values range from 1 to 7.

**Table 6:** Factor analysis for the 20-item Farmers’ Mindfulness and Life Satisfaction Scale (FMLSS) and internal reliability analysis.

Factor	Variance Explained	Cronbach’s Alpha	Items	Loadings
F1	18.5%	0.874	(12)	0.865
			(13)	0.923
			(14)	0.782
			(16)	0.789
F2	13.5%	0.767 or 0.839 without item (10)	(2)	0.879
			(3)	0.911
			(10)	0.701
F3	12.9%	0.703	(1)	0.796
			(11)	0.562
			(15)	0.723
			(19)	0.669
F4	11.6%	0.173 or 0.640 without item (9)	(6)	0.623
			(8)	0.781
			(9)	−0.695
			(20)	0.661
F5	10.7%	0.686	(5)	0.846
			(7)	0.843
F6	9.7%	0.658 or 0.672 without item (4)	(4)	0.593
			(17)	0.529
			(18)	0.903
Global FMLSS Cronbach’s alpha: 0.611 with all 20 items, or 0.607 without item (4), or 0.672 without item (9), or 0.588 without one item (10), or 0.655 without items (4), (9), and(10)				

**Table 7:** The mean scores and T-tests for each cluster considering the items individually and the three scales.

Item	Cluster 1 “Pleased Farmers” (*n* = 26) Mean ^1^ ± SD	Cluster 2 “Accommodated Farmers” (*n* = 4) Mean ^1^ ± SD	*p*-Value ^2^	Cohen’s d ^3^	Power of the Test ^4^
(1) I am aware that my emotions during harvesting can influence my thinking and behaviour	5.85 ± 1.61	1.90 ± 1.15	<0.001 *	1.566	0.804
(2) When something unexpected happens during harvesting, I am aware of my emotional state	5.75 ± 1.42	6.40 ± 0.69	0.384	1.360	0.686
(3) When something during harvesting doesn’t go well, I am aware of my inner frustration and restlessness	5.98 ± 1.16	6.10 ± 1.15	0.854	1.159	0.549
(4) When the situation changes during the harvesting, I am aware of the thoughts and ideas that flashed across my mind	5.52 ± 1.53	4.30 ± 2.47	0.180	1.657	0.846
(5) I find it difficult to stay focused on what’s happening in the present	4.00 ± 1.77	3.70 ± 2.66	0.769	1.887	0.924
(6) It seems I am ‘running on automatic’, without much awareness of what I’m doing	4.51 ± 1.69	3.70 ± 2.47	0.409	1.794	0.897
(7) I find myself doing things without paying attention	4.55 ± 1.67	5.20 ± 2.08	0.492	1.727	0.874
(8) I find myself listening to someone with one ear, doing something else at the same time	4.46 ± 2.02	4.60 ± 2.59	0.903	2.087	0.963
(9) I find myself preoccupied with the future or the past	2.98 ± 1.27	4.00 ± 2.86	0.224	1.519	0.779
(10) I snack without being aware that I’m eating	5.43 ± 2.03	6.70 ± 0.60	0.231	1.928	0.963
(11) In most ways my life is close to my ideal	5.46 ± 1.17	3.25 ± 0.96	0.001 *	1.153	0.545
(12) The conditions of my life are excellent	5.27 ± 1.00	4.00 ± 1.41	0.033 *	1.054	0.474
(13) I am satisfied with my life	5.69 ± 1.12	5.00 ± 2.16	0.321	1.275	0.630
(14) So far I have gotten the important things I want in life	6.15 ± 1.01	6.75 ± 0.50	0.260	0.966	0.412
(15) If I could live my life over, I would change almost nothing	5.00 ± 1.23	1.75 ± 0.96	<0.001 *	1.206	0.582
(16) Spending more time outdoors and in contact with nature makes me feel better	6.73 ± 0.53	6.25 ± 1.50	0.569	0.704	0.244
(17) I tend to worry about what other people think about me or what I do	2.65 ± 1.55	2.50 ± 1.29	0.852	1.522	0.781
(18) I often feel overwhelmed by the weight of responsibilities	3.81 ± 1.36	3.00 ± 1.63	0.288	1.389	0.704
(19) Farming makes me feel good	6.27 ± 0.87	4.50 ± 2.89	0.308	1.255	0.616
(20) I enjoy what I do, and I feel valued for it	5.58 ± 1.07	3.50 ± 2.38	0.179	1.273	0.629
Global scores for the 10 items of the MAAS	4.91 ± 0.67	4.66 ± 0.65	0.517	0.694	0.239
Global scores for the 5 items of the SWLS	5.51 ± 0.84	4.15 ± 1.05	0.006 *	0.861	0.340
Global scores for the 19 items of the FMLSS	5.19 ± 0.51	4.37 ± 0.59	0.007 *	0.520	0.155

^1^ Considering the previous normalization of all items to the same 7-point scale. ^2^ An asterisk indicates significant differences between clusters (*p* < 0.05). ^3^ Effect sizes, standardized. ^4^ Power = 1 − beta; varies between 0 and 1 (100% power).

**Table 8:** Sociodemographic characterization of the family farmer clusters.

Variable	Group	Pleased Farmers (%)	Accommodated Farmers (%)
Age	≤59 y	57.7	50.0
	60–69 y	15.4	0.0
	≥70 y	26.9	50.0
Gender	Male	69.2	25.0
	Female	30.8	75.0
Household	<3 persons	53.8	50.0
	≥3 persons	46.2	50.0
Marital status	Married	88.5	100.0
	Divorced or widowed	11.5	0.0
Education	Basic (1–9 y)	61.5	100.0
	Secondary (10–12 y)	11.5	0.0
	University degree	27.0	0.0
Parents were farmers	Yes	73.1	75.0
Professional training in agriculture	Yes	15.4	25.0
Is a fulltime farmer	Yes	34.6	50.0
Was always a farmer	Yes	19.2	25.0
Job situation	Employed	46.2	25.0
	Other	53.8	75.0
Area of the farm	[0; 0.25] ha	61.5	75.0
	]0.25; 0.5] ha	7.7	0.0
	]0.5; 0.75] ha	7.7	25.0
	]0.75; 1] ha	23.0	0.0
Weekly hours of farm work	<20 h	38.5	75.0
	20–40 h	46.2	25.0
	>40 h	15.3	0.0
Monthly income	EUR 665–865	26.9	50.0
	EUR 866–1365	30.8	0.0
	EUR >1366	42.3	50.0
Significance of agriculture for income	Residual (<25%)	50.0	75.0
	Low (25–50%)	38.5	25.0
	Medium (50–75%)	3.8	0.0
	High (>75%)	7.7	0.0
Alcoholic drinks	<1 time/month	15.4	25.0
	2–3 times/month	11.5	0.0
	2–3 times/week	19.2	25.0
	≥4 times/week	53.9	50.0
Daily hours of sleep	6–8 h	65.4	50.0
	>8 h	34.6	50.0
Participation in organizations, cultural associations, or events	None	65.4	100.0
	1–3 times/week	34.6	0.0
Go to associations for social engagement after work	None	57.6	75.0
	1–3 times/week	30.8	25.0
	4–6 times/week	11.6	0.0

## Data Availability

The original contributions presented in this study are included in the article. Further inquiries can be directed to the corresponding author.

## Appendix A

Table A1 presents the items of the scales as they were presented to the participants, i.e., in the original Portuguese language and the original measuring intervals, while Table A2 presents the final version of the FMLSS after translation into English and with all items in the same measuring interval that was used for validation.

**Table A1:** Questions as they were originally presented to participants, in Portuguese language.

Item	Quase Sempre						Quase Nunca
	1	2	3	4		5	6
1. Estou ciente de que as minhas emoções durante a colheita podem influenciar o meu pensamento e comportamento							
2. Quando algo inesperado acontece durante a colheita, estou consciente do meu estado emocional							
3. Quando algo não corre bem durante a colheita, estou consciente da minha frustração e inquietação interior							
4. Quando a situação muda durante a colheita, estou consciente dos pensamentos e ideias que me passaram pela cabeça							
5. Acho difícil manter o foco no que está a acontecer no presente							
6. Parece que estou a “funcionar em automático”, sem grande consciência do que estou a fazer							
7. Dou por mim a fazer coisas sem prestar atenção							
8. Dou por mim a ouvir alguém com um ouvido, a fazer outra coisa ao mesmo tempo							
9. Faço um lanche sem ter consciência de que estou a comer							
10. Na maioria dos aspetos, a minha vida está próxima do meu ideal							
	**Discordo Totalmente**						**Concordo Totalmente**
	**1**	**2**	**3**	**4**	**5**	**6**	**7**
11. As condições da minha vida são excelentes							
12. Estou satisfeito com a minha vida							
13. Até agora consegui as coisas importantes que quero na vida							
14. Se pudesse voltar a viver a minha vida, não mudaria quase nada							
15. Passar mais tempo ao ar livre e em contacto com a natureza faz-me sentir melhor							
16. Tenho tendência a preocupar-me com o que as outras pessoas pensam de mim ou com o que faço							
17. Muitas vezes sinto-me sobrecarregado pelo peso das responsabilidades							
18. A agricultura faz-me sentir bem							
19. Gosto do que faço e sinto-me valorizado por isso							

**Table A2:** Final version of questions in FMLSS, with uniform scoring on 7-point Likert scale.

Item	Almost Always						Almost Never
	1	2	3	4	5	6	7
1. I am aware that my emotions during harvesting can influence my thinking and behaviour							
2. When something unexpected happens during harvesting, I am aware of my emotional state							
3. When something during harvesting doesn’t go well, I am aware of my inner frustration and restlessness]							
4. When the situation changes during the harvesting, I am aware of the thoughts and ideas that flashed across my mind							
5. I find it difficult to stay focused on what’s happening in the present							
6. It seems I am ‘running on automatic’, without much awareness of what I’m doing							
7. I find myself doing things without paying attention							
8. I find myself listening to someone with one ear, doing something else at the same time							
	**Strongly Disagree**						**Strongly Agree**
	**1**	**2**	**3**	**4**	**5**	**6**	**7**
9. I snack without being aware that I’m eating							
10. In most ways my life is close to my ideal							
11. The conditions of my life are excellent							
12. I am satisfied with my life							
13. So far I have gotten the important things I want in life							
14. If I could live my life over, I would change almost nothing							
15. Spending more time outdoors and in contact with nature makes me feel better							
16. I tend to worry about what other people think about me or what I do							
17. I often feel overwhelmed by the weight of responsibilities							
18. Farming makes me feel good							
19. I enjoy what I do, and I feel valued for it.							
